# Metabolic diversity of Ferrovaceae and potential contributions to iron oxidation

**DOI:** 10.1128/aem.00700-26

**Published:** 2026-06-03

**Authors:** Christen L. Grettenberger, Jennifer L. Macalady, Trinity L. Hamilton

**Affiliations:** 1 Department of Earth and Planetary Sciences, University of California Davis 198844, Davis, California, USA; 2 Department of Environmental Toxicology, University of California Davis 115133, Davis, California, USA; 3 Department of Geosciences, Pennsylvania State University 171659, University Park, Pennsylvania, USA; 4 Department of Plant and Microbial Biology and the BioTechnology Institute, University of Minnesota 172728 , St. Paul, Minnesota, USA; Colorado School of Mines, Golden, Colorado, USA

**Keywords:** acid mine drainage, *Ferrovum*, bioremediation

## Abstract

Active and abandoned metal and coal mines generate acidic, metal-laden water that pollutes downstream areas, commonly referred to as acid mine drainage (AMD). AMD is host to microbial communities, including acidophilic iron oxidizers. Microbially mediated iron oxidation is a desirable (bio)remediation strategy for AMD. Ferrovaceae are Fe-oxidizing bacteria observed in AMD globally and thus could be an asset for bioremediation strategies. To better understand the potential for Ferrovaceae to contribute to AMD bioremediation, we analyzed 240 genomes and metagenome-assembled genomes from Ferrovaceae, including sequences from AMD sites with high iron oxidation rates. Based on our analyses, the phylogenetic and physiological diversity of this group is greater than previously known. We found that while all taxa are likely capable of iron oxidation using a cyc-2 like protein, some may also be capable of iron oxidation using an Mto-like protein. We also identified Ferrovaceae that are likely capable of anoxygenic phototrophy. Our findings indicate that multiple Ferrovaceae populations co-occur and suggest that differences in physiology may promote niche differentiation along resource axes. Physiologically diverse iron oxidizer communities could support a more resilient microbial community, resulting in higher iron oxidation rates and potentially more efficient bioremediation, and thus our results also indicate that future studies that link taxonomy with iron oxidation activity are warranted.

**IMPORTANCE:**

Acid mine drainage (AMD) pollutes watersheds worldwide. Microbial communities can be leveraged to improve AMD bioremediation because they drive biogeochemical processes in these ecosystems. In AMD streams, iron-oxidizing microbial populations remove iron from the AMD effluent by precipitating iron oxides, which absorb other metals. These communities vary across sites and differ in how rapidly they oxidize iron. The factors that contribute to iron oxidation rates are not well understood, making it difficult to design effective bioremediation strategies. Ferrovaceae populations are widespread in AMD globally, including in sites with exceptionally high rates of iron oxidation. To examine the potential for Ferrovaceae to be key components of bioremediation strategies, we examined the genomic content and functional potential of Ferrovaceae in publicly available metagenomic data sets. Our analysis uncovered several new species of Ferrovaceae as well as an expanded metabolic potential for this group. Comparative genomics suggests that functional diversity leads to co-occurrence of multiple Ferrovaceae species at the same sites. The presence of multiple iron-oxidizing taxa with distinct physiology could be beneficial for bioremediation strategies.

## INTRODUCTION

Acid mine drainage (AMD) is a global problem that pollutes tens of thousands of kilometers of streams worldwide (1–5). AMD is generated when mining activities expose iron-sulfide minerals in coal and other rocks to oxygen and water. This generates acidic, metal-laden water that kills benthic macroinvertebrates, fish, and other aquatic species. Acid mine drainage remediation strategies are costly and often include regular intervention with heavy machinery and/or large infrastructure (e.g., active remediation via aeration, lime addition, or bioreactors) (6). In contrast, bioremediation occurs in more cost-effective passive treatment requiring only minimal continued inputs. One such passive treatment method leverages the metabolisms of naturally occurring iron-oxidizing species like *Acidithiobacillus ferrooxidans*, *Gallionella ferruginea*, and *Ferrovum myxofaciens* to oxidize iron, effectively removing it from the stream environment (6). Although iron-oxidizing species contribute to AMD formation from iron sulfide minerals in the subsurface, they play a different role in metal-rich surface AMD, where iron-sulfide minerals are absent. In these environments, iron oxidizers remove reduced iron from solution, making downstream treatment less costly. For example, passive treatment systems frequently employ limestone beds that become clogged or armored with iron oxides, preventing them from neutralizing further acids and necessitating frequent intervention (6). Microbial iron oxidation can remove iron from AMD solutions before neutralization, making limestone beds and other active and passive treatment structures more effective and cheaper to maintain.

Previous studies have documented significant variation in iron oxidation rates at AMD sites. For example, zero-order iron oxidation rates vary by nearly an order of magnitude in Appalachian coal mine drainages and by approximately 5× in the Iberian Pyrite Belt (7). These differences are likely due, in part, to thermodynamics—the Gibbs free energy available from iron oxidation is higher at low pH than it is at higher pH (8). However, thermodynamics cannot fully account for differences in iron oxidation rates. Sites in the Iberian Pyrite Belt had lower iron oxidation rates than those in Appalachian coal mine drainages, even when the Gibbs free energy available was greater (7, 8), and laboratory chemostats did not replicate field oxidation rates even with geochemical setpoints designed to replicate *in situ* conditions (8, 9). Thus, there is another controller on iron oxidation rates in AMD in the field.

To leverage microbial iron oxidation for bioremediation, we must understand the physiology of iron-oxidizing organisms and the metabolic and ecological factors that correlate with high iron oxidation rates. Microbial taxa differ in the proteins used to oxidize iron (to transfer electrons from reduced iron (Fe(II)) to the cell) (10), which likely impacts iron oxidation rates. All known iron oxidizers use one or more of three iron-oxidizing protein types: Cyc2-like proteins (11, 12), MtoA, or its homolog PioA (13–15). Other physiological adaptations can also impact iron oxidation rates. For example, the ability to fix nitrogen may increase niche breadth to nitrogen-limited environments. At the same time, nitrogen fixation is inhibited by oxygen (16), and many Fe(II) oxidizers are microaerophiles that use oxygen as an electron donor (e.g., *Ferrovum*) (17, 18). Iron-oxidizing taxa with motility may move to ideal redox conditions as environmental conditions fluctuate across the day/night cycle, resulting in higher net iron oxidation rates compared to immobile taxa. Transcripts mapping to flagellar genes in the Fe(II)-oxidizing taxon *Ferrovum* were recovered from AMD sediments (18), but specific taxis was not investigated. Community-level interactions also likely impact iron oxidation rates. For example, there is evidence to suggest that the presence of multiple Fe(II)-oxidizing species provides functional redundancy and stabilizes Fe(II) oxidation rates in bioreactors (19). Additional evidence for community interactions, including carbon transfer, has been proposed based on the observations of stable co-cultures of iron-oxidizing taxa and heterotrophic bacteria (20, 21).

Ferrovaceae is an iron-oxidizing group widely distributed in AMD ecosystems globally (3, 5, 21–32) and contains the genus *Ferrovum*. In AMD, *Ferrovum* are typically recovered from sites with pH 2–4 and Fe(II) concentrations ranging from 100 to 400 mg/L Fe(II) (5, 17, 25, 33). In laboratory and field studies, *Ferrovum* has been implicated in active Fe(II) oxidation, including the highest rates of iron oxidation reported (3, 7, 9, 31, 32, 34, 35). For example, *Ferrovum* and *Gallionella* were abundant in a pilot plant designed to treat AMD across seasonal fluctuations in mine water flow, temperature, etc. (32). Furthermore, in a continuous flow reactor at pH ~2 with 280 mg/L Fe(II), where *Ferrovum* is the only Fe oxidizer present, ~90% of Fe(II) was oxidized over the course of the experiment (34). Therefore, this genus may play a pivotal role in AMD biogeochemical cycling and bioremediation worldwide.

Multiple clades of Ferrovaceae are represented in sequence databases, and genomes and metagenome-assembled genomes have significant functional gene diversity (17, 20), including among populations from the same site and same sample (27). As mentioned above, species within *Ferrovum* differ in their ability to fix nitrogen and the presence of genes for flagella (21, 27, 36–38). These differences may influence iron oxidation rates. Unfortunately, *Ferrovum* is difficult to cultivate, and only the type species *Ferrovum myxofaciens* P3G has been isolated (17), limiting culture-based approaches to examine the potential for this group to facilitate AMD bioremediation. (Meta)genomic approaches can provide insights into the potential metabolic breadth of Ferrovaceae, informing both *in situ* studies and culture-based approaches.

The genome content of *Ferrovum* has been shaped by gene transfer events and genome reduction in some taxa (36) and, therefore, is not congruent with the evolutionary history of the genus. In order to understand the diversity of physiological traits in Ferrovaceae taxa and how these traits relate to their phylogeny, we generated metagenome-assembled genomes (MAGs) of Ferrovaceae spp. from Scalp Level Run (SLR), PA, a site with the highest measured iron oxidation rate in the Appalachian coal belt and Iberian Pyrite Belt (7), with *Ferrovum* spp. as a major component of its microbial community (3). We compared the genome content of SLR *Ferrovum* genomes to that of nearly 200 publicly available MAGs and genomes of populations within the family Ferrovaceae to investigate the traits of SLR *Ferrovum* spp. that might confer high iron oxidation rates and whether these traits are globally distributed.

## MATERIALS AND METHODS

### Scalp Level metagenomics

A sediment sample was collected from near the emergence of Scalp Level Run, PA (40.25;−78.84). Approximately 3 g of surface sediment was collected in a sterile tube, preserved with 15 mL of RNALater, transported on ice, and stored at −20°C. DNA was extracted using a MoBio PowerBiofilm DNA extraction kit (MoBio, Carlsbad, USA). Each sample was extracted twice with a 2-min and 4-min vortex adapter bead beating time, and the products were pooled. Libraries were prepared using the Nextera XT Library Preparation kit following the manufacturer’s instructions. Paired-end 2 × 150 bp sequencing was performed using an Illumina HiSeq 2500. Reads were quality-checked using FastQC 0.11.9 (39). Trimmomatic 0.39 was used to remove leading and trailing low-quality and N bases, cut the sequence when the average quality drops below 15, and remove reads shorter than 36 bases long (40). Reads were assembled using megahit 1.0.6 with a minimum contig length of 1,500 bp (41). Reads were mapped using samtools 1.15.1 and bowtie2 (42, 43). The jgi-summarize_bam_contig_depths command in MetaBAT 2.12.1 was used to calculate read depth, and MetaBAT was used to bin contigs longer than 2,500 bp (44). Bins were classified using GTDB-Tk 2.1.0 (45). The completeness and contamination for each bin were calculated by CheckM 1.0.13 (46). Bins classified as belonging to Ferrovaceae were retained for additional analysis.

### Pangenomics and phylogenetics

We searched the NCBI SRA database for all MAGs identified as Ferrovaceae and retrieved those MAGs (25 September 2024). Seven genomes identified as *Gallionaceae* spp. were included to provide a root for phylogenetic analyses. Genome completeness and contamination were calculated using CheckM 1.0.13 (46). Because there are disagreements between NCBI and GTDB taxonomies, GTDB-tk 2.1.0 (45) was used to re-classify the MAGs and genomes. Genomes and MAGs that were classified as Ferrovaceae by both GTDB and NCBI and were more than 75% complete with less than 5% contamination and all MAGs from Scalp Level Run were retained. Genomes were classified into genera or species based on the GTDB-tk-generated taxonomy. Genera were re-named from the GTDB placeholder name following the International Code of Bacterial Nomenclature. Names for uncultivated genera are *Candidatus*.

Anvi’o v8 was used to generate a contigs database for the Ferrovaceae bins using the anvi-gen-contigs database command (47). The command anvi-run-kegg-kofams annotated putative protein-coding genes using the KOfam database (48). Anvi-estimate-metabolism was used to determine the completeness of metabolic pathways including carbon fixation, sulfur oxidation, nitrogen fixation, and anoxygenic photosynthesis. It was also used to determine which flagellar biosynthesis genes were encoded in the MAGs. Because genomes either contained few (i.e., <7) or many (>30 out of a potential 54) genes, genomes were considered to encode the genes necessary for flagellar assembly if they contained >30 of the genes in pathway number 20240 (flagellar assembly). The anvi-run-hmms command identified hits in the bacteria71 hmm collection, a curated set of single-copy genes from GToTree (49). These genes include the following: ADK, AICARFT_IMPCHas, ATP-synt, ATP-synt_A, Adenylsucc_synt, Chorismate_synt, EF_TS, Exonuc_VII_L, GrpE, Ham1p_like, IPPT, OSCP, PGK, Pept_tRNA_hydro, RBFA, RNA_pol_L, RNA_pol_Rpb6, RRF, RecO_C, Ribonuclease_P, Ribosom_S12_S23, Ribosomal_L1, Ribosomal_L13, Ribosomal_L14, Ribosomal_L16, Ribosomal_L17, Ribosomal_L18p, Ribosomal_L19, Ribosomal_L2, Ribosomal_L20, Ribosomal_L21p, Ribosomal_L22, Ribosomal_L23, Ribosomal_L27, Ribosomal_L27A, Ribosomal_L28, Ribosomal_L29, Ribosomal_L3, Ribosomal_L32p, Ribosomal_L35p, Ribosomal_L4, Ribosomal_L5, Ribosomal_L6, Ribosomal_L9_C, Ribosomal_S10, Ribosomal_S11, Ribosomal_S13, Ribosomal_S15, Ribosomal_S16, Ribosomal_S17, Ribosomal_S19, Ribosomal_S2, Ribosomal_S20p, Ribosomal_S3_C, Ribosomal_S6, Ribosomal_S7, Ribosomal_S8, Ribosomal_S9, RsfS, RuvX, SecE, SecG, SecY, SmpB, TsaE, UPF0054, YajC, eIF-1a, ribosomal_L24, tRNA-synt_1d, and tRNA_m1G_MT. Genes were aligned and then concatenated. The resulting concatenated sequences were used to construct a phylogenetic tree. IQtree v2.3.1 (50) was used to select the best model for protein substitution and build a consensus tree using 1,000 bootstrap replicates for both nonparametric bootstraps and for SH-aLRT. The tree was visualized using ggtree (v3.19) and ggtreeExtra (v3.19) (51). FeGenie v1.2 (52) identified iron-related genes in the genomes. Because the Mto, Mtr, and Pio genes are often homologous, we retrieved any contigs identified by FeGenie as containing these genes. We then used the online basic local alignment search tool (BLAST v 2.16.0) (53) to search for close relatives of the translated amino acid sequences.

Hidden Markov models (HMMs) and amino acid sequences for the PufL and PufM reaction center proteins were retrieved from EggNog 5.0 (54). We used these HMMs to identify, retrieve, and translate the genes encoding PufL and PufM using the anvi-run-hmms and anvi-get-sequences-for-hmm-hits commands in Anvi’o using a cutoff value of 1E-15. The sequences for PufL and PufM retrieved from EggNog and those from the genomes were aligned using MAFFT v7.505 using the –auto option (55). The model selected by the software was L-INS-i. The alignment was trimmed with trimAl v1.5.0 (56) using the automated1 method. A phylogenetic tree was built using IQtree v2.3.1 (50), as described above. The trees were visualized in iTOL and rooted at the midpoint for visualization purposes (57).

### Biogeography of phototrophic Ferrovaceae species

We used sourmash branchwater (58) to determine the geographic distribution of phototrophic Ferrovaceae. The representative genome for each species was uploaded to the branchwater metagenome query site hosted by the Joint Genome Institute (https://branchwater.jgi.doe.gov). Branchwater uses sourmash (59, 60) to search 1.2 million publicly available metagenomes and identify those that contained these species. To ensure that we were looking only at the hits within the species, we removed all hits with an average nucleotide identity (ANI) less than 97%.

## RESULTS

We retrieved three MAGs from the Scalp Level metagenome. These were 65%, 74.9%, and 88% complete with 0%–1.2% contamination in 160–316 contigs (Table S1). We retrieved 240 MAGs identified as Ferrovaceae from the NCBI SRA. Sixty-one genomes were removed for being less than 75% complete or having more than 5% contamination. One of those genomes was also not identified as Ferrovaceae by GTDB-tk. The final data set included 179 Ferrovaceae genomes or MAGs from NCBI, three MAGs from Scalp Level Run, and the seven Gallionaceae genomes used as an outgroup. From the Ferrovaceae genomes, we identified 22 unique species in seven genera.

### Diversity of Ferrovaceae

**Fig 1:**
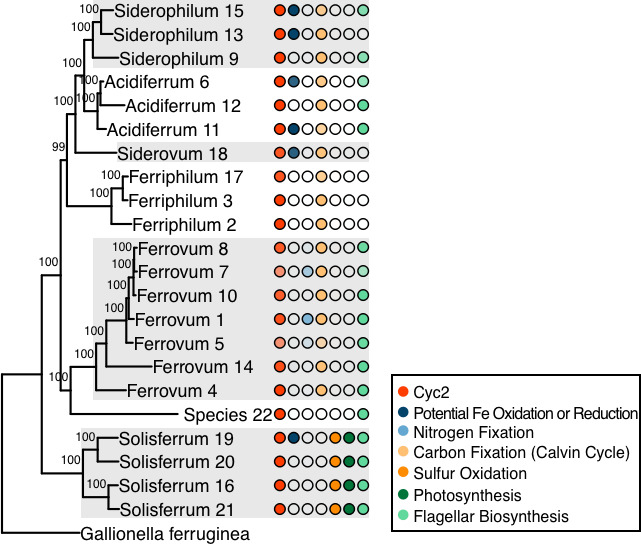
Phylogeny of Ferrovaceae taxa based on the concatenated marker gene tree. Bootstrap values for 1,000 bootstrap replicates are indicated at the node. The number of genomes present in the taxon is indicated in parentheses. Metabolic potential is indicated by symbols on the leaves. The transparency of the color indicates the number of genomes within the species that encode that metabolic process. The tree was built using the Q.insect+F+R5 substitution model. For visualization purposes, only a single leaf for each species is shown.

The MAGs and genomes clustered into four main clades (Fig. 1) based on the concatenated single-copy gene tree. Clade 1 is an outgroup to all other Ferrovaceae species and includes Species 16, 19, 20, and 21. Clade 2 includes eight species (Species 22, 4, 14, 5, 1, 10, 8, and 7) and is an outgroup to the third and fourth clades. The third clade is composed of Species 2, 17, and 3. The final clade is composed of seven species (Species 18, 9, 13, 15, 11, 12, and 6). Based on classifications from the Genomes Taxonomy Database (61) Ferrovaceae comprises seven genera. They also differ in their metabolic potential. We describe these species, propose names for candidate genera, and describe their metabolic potential below. The SLR MAGs are in Species 22, 1, and 7 (Clade 2).

#### Clade 1

**Fig 2:**
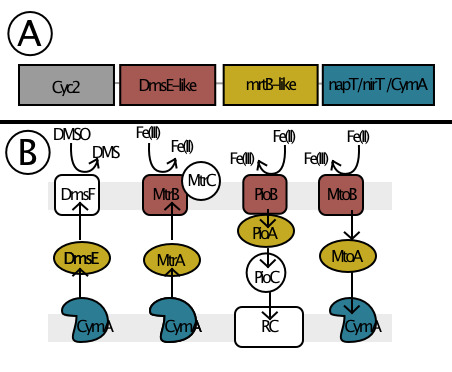
(**A**) Genes found in Ferrovaceae genomes that encode proteins that may be involved in Fe(II) oxidation or Fe(III) reduction. (**B**) Pathways for dimethyl sulfoxide (DMSO) and Fe(III)reduction via the Mtr pathway and Fe(II) oxidation via the Pio and Mto pathways. Proteins are color-coded to match the potential homolog found in the genome (illustrated in A). No shading indicates that genes encoding a homolog of this protein were not found.

The first clade of Ferrovaceae comprises four species (Species 16, 19, 20, and 21) from a single genus (Fig. 1; Fig. S1). These genomes were recovered from high-latitude, stratified lakes. Species 19 and 20 were found exclusively in stratified lakes in Alaska, USA, where the pH is between 4.4 and 5, the concentration of ferrous iron is between 3.3 and 54.6 mg/L, and the concentration of dissolved oxygen (DO) is between 8.01 and 9.02 mg/L (62). Species 21 was found in stratified lakes in Sweden and Finland. Only one location had geochemical data available, and the pH was 5.6, the concentration of Fe(II) was 21.1 mg/L, and the DO was 5.6 mg/L (62). Species 16 was found in the same lake in Finland as Species 21, and no geochemical data are available for that lake. All members of this clade contain genes encoding the photosystem II reaction center proteins PufL and PufM, Fe(II) oxidation using Cyc2, and sulfur oxidation from thiosulfate to sulfate (soxA, B, C, D, X, Y, and Z). Species 19 also contains a region that encodes a DMSE-family decaheme *c*-type cytochrome, an MtrB/PioB -family decaheme-associated outer membrane protein, an NapC/NirT-like protein, and another *c*-type cytochrome (Table 1; Fig. 2). All species encode the genes necessary for flagellar biosynthesis. The Genomes Taxonomy Database (GTDB) (61) currently uses the placeholder name CAIVHB01 for this genus. We propose the name “*Candidatus Solisferrum*” gen. nov. (so.lis.fer′rum. L. n. sol, the sun; L. n. ferrum, iron; N.L. neut. n. Solisferrum, referring to the putative ability of this genus to perform phototrophy and to oxidize iron).

**TABLE 1:** Metabolic potential of Ferrovaceae taxa^*a*^

Clade	Species number	No. of genomes	Genus	Carbon fixation	Fe(II) oxidation	Fe(II) oxidation or Fe(III) reduction	Nitrogen fixation	Sulfur oxidation	Anoxygenic phototrophy	Flagella
1	21	1	Ca. *Solisferrum*		**100**			**100**	**100**	**100**
1	20	3	Ca. *Solisferrum*		**100**			**100**	**100**	**100**
1	19	3	Ca. *Solisferrum*		**100**	**100**		**100**	**100**	**100**
1	16	1	Ca. *Solisferrum*		**100**			**100**	**100**	**100**
2	22	1	N/A	*	**100**					**100**
2	4	1	*Ferrovum*	**100**	**100**					**100**
2	14	37	*Ferrovum*	**94**	**89**	*3*	*3*			**100**
2	5	16	*Ferrovum*	50	50		*18*			**93**
2	1	9	*Ferrovum*	**100**	**88**		**77**			**100**
2	10	5	*Ferrovum*	**100**	**80**					**100**
2	8	8	*Ferrovum*	**100**	**83**		*8*			**92**
2	7	2	*Ferrovum*	50	50		**100**			**100**
3	2	9	Ca. *Ferriphilum*	**100**	**100**					
3	17	8	Ca. *Ferriphilum*	**75**	**88**					
3	3	5	Ca. *Ferriphilum*	**100**	**100**					
4	18	46	Ca. *Siderovum*	**91**	**84**	**82**				
4	9	7	Ca. *Siderophilum*	**100**	**100**					71
4	13	1	Ca. *Siderophilum*	**100**	**100**	**100**				**100**
4	15	4	Ca. *Siderophilum*	**75**	**100**	**100**				**75**
4	11	3	Ca. *Acidiferrum*	67	**100**	**100**				**100**
4	12	2	Ca. *Acidiferrum*	**100**	**100**					100
4	6	6	Ca. *Acidiferrum*	**83**	**100**	**80**				67

^*a*^ The percentage of genomes in the species that contain the genes necessary for the metabolic process are indicated in each cell. A single asterisk (*) indicates that the genome for this clade was incomplete (<65%). Therefore, absence in the genome may be due to incompleteness of the MAG. Cells are coded based on the number of genomes within the species that encoded the pathway, with 75%–100% indicated by bold font, between 25% and 75% by regular font, and less than 25% in italics. Empty cells indicate that no members of the species encode the genes necessary for the metabolic process. Grey shading indicates the presence of the gene in at least one MAG.

#### Clade 2

The second clade of Ferrovaceae contains eight species (Species 22, 4, 14, 5, 1, 10, 8, and 7) from two genera, including the type strain *Ferrovum myxofaciens* P3G in the genus *Ferrovum* (Species 4, 14, 5, 1, 10, 8, and 7) and n MAG from SLR (Wind_5; Species 22). The SLR MAG is only 65% complete, which might confound accurate phylogenetic placement. Therefore, we decline to name this genus or interpret its metabolic potential until a better genome is available.

Species 1 is found in AMD sites in Wales, Germany, Slovakia, the Czech Republic, and the United States (9, 17, 19, 21, 63). Species 4, 7, and 22 are thus far only found in samples from Scalp Level Run—either from the sediments themselves or from bioreactors inoculated with those sediments (19, 64). Species 5 is also found at Scalp Level Run and in AMD sites in China. Species 8 is found at AMD sites in China and in the United States (65, 66). Species 10 and 14 are found exclusively at AMD sites in China. Members of the genus *Ferrovum* are capable of carbon fixation via the reductive pentose phosphate cycle and Fe(II) oxidation with Cyc2. Some members of Species 1, 5, 7, 8, and 14 are capable of nitrogen fixation (Table 1). All species within the genus *Ferrovum* appear to encode the genes for flagellar biosynthesis.

#### Clade 3

The third clade of Ferrovaceae contains three taxa (Species 2, 17, and 3) from mining waste and AMD sites across Canada, Europe, and China. Species 2 is found in AMD sites in Canada and Germany (67), Species 3 in AMD sites in Germany and China (21, 65), and Species 17 is found exclusively in AMD sites in China. This clade is composed of a single genus. Members of this clade are capable of carbon fixation via the reductive pentose phosphate cycle and Fe(II) oxidation with Cyc2. None of the species encodes the genes necessary for flagellar biosynthesis. This genus includes the named species *Ferrovum* sp. PN-J185 (Species 2) for which the genus name is incorrect. We propose that this species be renamed *Ferriphilum* sp. PN-J185 (see below).

GTDB currently uses the placeholder name PN-J185 for this genus. We propose the name “*Candidatus Ferriphilum*” gen. nov. (fer.ri.’phi.lum. L. n. ferrum, iron; Gr. Adj. philos, loving; N.L. neut. n. Ferriphilum, referring to the preference of this genus for iron-containing environments and putative ability to perform iron oxidation).

#### Clade 4

The final clade of Ferrovaceae contains seven species (Species 18, 9, 13, 15, 11, 12, and 6) and includes taxa from stratified lakes in Finland and Sweden (Species 18) (62) and AMD sites in China and the United States (Species 9, 13, 15, 11, 12, and 6) (65, 66, 68). Based on GTDB-tk results, this clade contains three genera currently indicated by GTDB placeholder names CAIVUX0 (Species 18), JAKBAT01 (Species 9, 13, and 15), and JAJZRV01 (Species 6, 11, and 12). Species 18 is found exclusively in stratified lakes in Finland and Sweden with pH between 4.6 and 6.1, dissolved Fe(II) from below the detection limit to 31.5 mg/L, and DO between nondetectable and 7.71 mg/L (59). Species 9, 13, and 15 are found exclusively in AMD sites in China (65). Species 6 was found exclusively in an AMD site in VT, USA (66, 68), and Species 11 and 12 were found exclusively in AMD sites in China (65). Members of this group are capable of carbon fixation via the reductive pentose phosphate cycle and Fe(II) oxidation with Cyc2. Six taxa (Species 18, 13, 15, 11, and 6) contain a region that encodes for a DMSE-family decaheme *c*-type cytochrome, an MtrB/PioB -family decaheme-associated outer membrane protein, an NapC/NirT-like protein, and another *c*-type cytochrome (Table 1). Some members of all species, except Species 18, encode the genes necessary for flagellar biosynthesis.

To replace placeholder name CAIVUX0 (Species 18), we propose the name “*Candidatus Siderovum* gen. nov” (sid.er.o’vum Gr. N. sideros, iron; L.n. ovum, egg; N.L. neutr. N. Sideovum, “iron egg,” referring to the preference of this taxon for iron-rich environments and referencing the cell shape described in the *Ferrovum* type species).

To replace placeholder name JAKBAT01 (Species 9, 13, and 15), we propose the name “*Candidatus Siderophilum*” gen. nov. (sid.er.o’.phil.um Gr.n sideros, iron; Gr. Adj. philos, loving; N.L. neut. n. Siderophilum, referring to the preference of this genus for iron-containing environments and putative ability to perform iron oxidation).

To replace placeholder name JAJZRV01 (Species 6, 11, and 12), we propose the name “*Candidatus Acidiferrum* gen. nov.” (acidi.ferr’um ; L.adj. acidus, acidic; L.n. ferrum, iron, referring to the preference of this organism for acidic environments and its putative ability to perform iron oxidation).

### Anoxygenic photosynthesis

**Fig 3:**
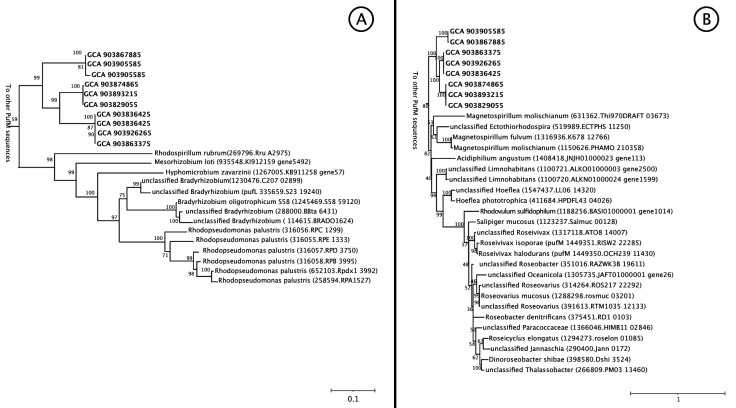
Phylogeny of (**A**) PufL and (**B**) PufM translated nucleotide sequences. Bootstrap values for 1,000 bootstrap replicates are indicated at the node. Trees were rooted at the midpoint for visualization purposes. Sequences from each taxon are indicated in bold, with the accession number for the genome in parentheses. The substitution model for PufL was LG+F+R5, and for PufM, it was LG+R6.

Four species of Ferrovaceae, those within the genus Ca. *Solisferrum*, encode genes for anoxygenic photosynthesis via photosystem II, including the PufL and PufM reaction center subunits; PufC, the reaction center cytochrome C subunit; and bacteriochlorophyll-related genes *bchD*, *E*, *I*, *L*, *M*, *X*, *Y*, and *Z*, and are therefore likely capable of anoxygenic photosynthesis. The translated sequences from these taxa form a monophyletic group in *pufL* and *pufM* phylogenies (Fig. 3) with strong bootstrap support (bootstrap support = 99 for PufL and 100 for PufM). The closest relatives to the PufL sequences are from the alphaproteobacterial genera *Rhodopseudomonas*, *Bradyrhizobium*, *Hypohomicrobium*, *Mesorhizobium*, and *Rhodospirillum*, but this node has weaker bootstrap support (bootstrap = 59) (Fig. 3A) The closest relatives to the PufM sequences are from the alphaprotobacterial genera *Magnetospirillum*, *Acidiphilium*, *Hoeflea*, *Rhoduvulum*, *Salipiger*, *Roseivivax*, *Roseobacter*, *Oceanicola*, *Roseovarius*, *Paracoccaceae*, *Roseicyclus*, *Jannaschia*, *Dinoroseobacter*, and *Thalassobacter*; the betaproteobacterial order *Limnohabitans*; and gammaproteobacterial genus *Ectothiorhodospira* (Fig. 3B). The overall phylogenetic placement of these groups was retained in a tree that was constructed with both PufL- and PufM-encoding genes (data not shown).

## DISCUSSION

### Diversity of Ferrovaceae

The diversity of Ferrovaceae is higher than that previously reported (21, 27, 36), both phylogenetically and metabolically. Our analysis shows that Ferrovaceae comprises four clades with seven genera and 22 species. Common metabolic traits include iron oxidation using the Cyc-2-like protein and carbon fixation via the reductive pentose phosphate pathway. However, Ferrovaceae taxa differ in their ability to fix nitrogen, oxidize thiosulfate, and perform anoxygenic phototrophy. Some taxa also have a suite of genes that may encode an alternative pathway for iron oxidation or a pathway for iron reduction (see “Iron cycling,” below). Definitively demonstrating the function of the proteins encoded in this pathway requires a robust culture-based approach.

#### Anoxygenic phototrophy

Four Ferrovaceae species (Species 16, 19, 20, and 21) encode genes for the PufL and PufM photosystem II proteins in addition to the genes necessary for the production of bacteriochlorophyll. The reaction center sequences are monophyletic and closely related to sequences from Alpha- and Gammaproteobacteria, suggesting that their shared ancestor acquired the genes for these proteins via a single horizontal transfer event. These genomes lack the genes necessary for carbon fixation via the Calvin cycle. However, this may be due to the incomplete nature of the MAGs. The capacity for anoxygenic phototrophy has not been reported in Ferrovaceae before but is common in Proteobacteria, including in Burkholderiales, close relatives of Ferrovaceae. Because these putatively phototrophic Ferrovaceae are capable of iron oxidation and thiosulfate oxidation, it is likely that they either use thiosulfate or Fe(II) as an electron donor in anoxygenic phototrophy. Photoferrotrophy is reported in a wide variety of groups, including Proteobacteria (e.g., *Rhodovulum*, *Rhodopseudomonas*, and *Rhodobacter* spp.) and Chlorobi (e.g., *Chlorobium* spp.) (69–71). In these known photoferrotrophs, PioA/B catalyzes iron oxidation (14, 15). However, pioA and pioB genes were not present in Species 16, 19, 20, and 21. The use of sulfide or thiosulfate as an electron donor for anoxygenic photosynthesis is common in the green and purple sulfur bacteria (Chlorobi, Chromatiaceae, and Ectothiorhodospiraceae) (72). The lack of PioA/B and presence of sulfur oxidation genes (sox) in Species 16, 20, and 21 suggest that they use thiosulfate rather than Fe(II) as an electron donor for phototrophy. Although Species 19 contains a potential homolog of pioB, it is likely that this gene encodes MtoB rather than PioB (see below).

The four taxa capable of anoxygenic phototrophy inhabit stratified freshwater lakes in high latitudes (62). Based on samples with available site descriptions, these taxa reside at depths that are putatively in the photic zone (0–0.7 meters), at pH values 4.4–5.6 with 3.3–54.6 mg/L Fe(II), and in well-oxygenated waters (DO between 5.6 and 9.02) (62). At the 97% similarity level, Branchwater did not identify any additional environments where these taxa are found, including AMD. Because Branchwater finds all instances of target taxa in publicly available data sets (>1 million metagenomes), these members of Ferrovaceae appear to be unique. These results suggest that anoxygenic phototropic *Ferrovum* may be geographically restricted, dispersal-limited, and/or rare (low abundance) and thus not typically recovered in metagenomic surveys.

#### Iron cycling

Iron-oxidizing taxa use outer membrane proteins to oxidize Fe(II). All known acidophilic iron oxidizers oxidize iron using cytochrome *c* in the outer membrane, and the electrons are passed on through the periplasm and inner membrane using a variety of redox proteins (10). Neutrophilic iron oxidizers have a wider variety of iron-oxidizing complexes, including MtoA and its homolog PioA, which also oxidize iron in the outer membrane (13). Based on our analyses, Ferrovaceae may differ in their strategies for iron oxidation. There are three clusters of Cyc2 proteins used in iron oxidation. The first contains Cyc2 sequences from neutrophilic iron oxidizers, the second contains those from *Acidithiobacillus ferrooxidans*, and the third contains those from *Leptospirillum ferrooxidans* (52). All recovered Ferrovaceae MAGs encode the genes for Cyc2 iron oxidation using a cluster 2 Cyc2-like protein. This is consistent with the observation that members of the genus *Ferrovum* inhabit a pH niche more similar to that of *Acidithiobacillus* than *Leptospirillum* or neutrophilic Fe(II) oxidizers (5, 25, 33). Cyc2 is likely adapted to specific environmental and thermodynamic conditions, including the redox potential of Fe(II)/Fe(III) (12, 73–75), oxygen concentration, and the solubility of Fe(III) minerals (which play a role in Fe(II) oxidation kinetics) (7).

A subset of species also has a series of putative iron cycling-related genes that encode a c-type cytochrome, a DmsE-family decaheme outer membrane protein, an MtrB/PioB family protein, and an NapC/NirT family cytochrome c (Fig. 2). We predict that this series of genes encodes an electron transfer pathway that may be used for iron oxidation or reduction, but it is difficult to determine the roles of the proteins they encode using sequence-based analyses alone. The NapC/NirT family of proteins, including CymA and ImoA, is used to transfer electrons between the quinol pool and periplasmic proteins (76–78). While these are often associated with reductive systems—for example, the reduction of nitrate, Fe(III), and arsenic (77, 78)—one protein, ImoA, is used in the oxidation of reduced iron (79).

The DmsE family of decaheme c-type cytochromes, including the paralogs MtrA and MtoA/PioA, transfer electrons across the periplasm. In DMSO respiration, DmsE transfers electrons via DmsF to the DmsAB reductase complex (80, 81). In Fe(III) reduction, MtrA interacts with MtrB and extracellular terminal reductases like MtrC or OmcA (82–84). During Fe(II) oxidation, MtoA or PioA interfaces with the porins MtoB or PioB, respectively, forming an outer-membrane conduit for electrons derived from extracellular Fe(II) and transferring them inward toward the quinol pool via inner-membrane NapC/NirT-family proteins (13–15, 79). Because it is difficult to determine the function of these types of multiheme cytochromes using sequence-based analyses or homology (52), it is not clear what function this set of genes is used for. DMSO respiration requires DmsF, DmsA, and DmsB (80), which we did not identify in this region. Similarly, Fe(III) reduction with Mtr requires MtrC, an outer membrane decaheme (82–84) that was not identified in the same neighborhood as these genes, suggesting that these species do not have the Mtr pathway. Pio has thus far exclusively been described in photoferrotrophic organisms (14), and most of the Ferrovaceae containing these genes do not have photosynthetic reaction center proteins. Additionally, the Pio iron oxidation pathway requires PioC, a high-energy iron-sulfur protein (14), which we did not identify in this gene neighborhood (Fig. 3B). Therefore, we hypothesize that this region encodes for an Mto-like pathway similar to that found in the neutrophilic Fe(II) oxidizer, *Sideroxydans lithotrophicus* (13, 79). Future culture-based approaches could be used to test this hypothesis.

If some members of the Ferrovaceae encode both the Cyc-2-like and Mto pathways for iron oxidation, they may be used under different geochemical or environmental conditions. The Cyc2 complex is smaller than Mto and is predicted to have a single heme (12). Therefore, synthesis and maintenance of the Cyc2 complex might require fewer resources (12). However, Mto can oxidize mineral-bound Fe(II) (79) in addition to dissolved Fe(II), suggesting that the taxa that encode Mto could access particulate or solid Fe(II). Taxa with MtoA could be at a competitive advantage in environments with limited dissolved Fe(II).

### Contribution of Ferrovaceae to rapid iron oxidation at Scalp Level Run

Fe(II) oxidation rates at SLR are high relative to other measured locations, making it an attractive site for examining the biotic and abiotic factors that contribute to high rates. The Fe(II) oxidation rate is likely determined in part by the composition of the microbial community as well as pH and oxygen and Fe(II) concentrations (25, 33). At SLR, environmental conditions are similar to those in field and bioreactor studies where abundant *Ferrovum* are observed in tandem with high rates of Fe(II) oxidation (e.g., pH at SLR is 2.77–2.96). And, at SLR, *Ferrovum* accounts for 49%–63% of the microbial community. The correlation between high *Ferrovum* abundance and high iron oxidation rate could be due to biotic factors such as gene content or community structure or due to interactions and feedbacks among physical, chemical, and biological parameters. Thus, like other field and bioreactor studies where *Ferrovum* is abundant (or the only Fe(II) oxidizer), *Ferrovum* may contribute to high Fe(II) oxidation rates at SLR (3). However, this assumption requires explicit data on *Ferrovum* Fe(II) oxidation rates.

Rates of Fe(II) oxidation in SLR sediments were lower in bioreactor studies compared to those measured *in situ* (9). *In situ*, three *Ferrovum* taxa were recovered (Species 1, 5, and 7), while only Species 5 and 7 were recovered in the bioreactors (19, 64). Taxa 1, 5, and 7 are members of the second Ferrovaceae clade (Fig. 1) and have similar metabolic potential. Increased diversity (both intra- and interspecies) increases the number of ecosystem functions and services provided by a microbial community in terrestrial ecosystems (85), while environmental heterogeneity can create multiple niches, including on microscopic spatial scales (86). In AMD, intraspecies diversity and functional redundancy in *Ferrovum* could contribute to stable and rapid Fe(II) removal. While Species 1, 5, and 7 from SLR encode Fe(II) oxidation pathways (functional redundancy), Species 1 and 7 have the potential to fix nitrogen and use flagella for motility, suggesting niche differentiation. The increased diversity of Ferrovaceae in SLR may enable the iron-oxidizing community to oxidize iron in different niches, under different geochemical microenvironments, or at different times of the day (e.g., as the concentration of oxygen varies based on the activity of eukaryotic phototrophs).

At SLR, members of Ferrovaceae predominate, making up >50% of the microbial community (3). However, *Ferrovum* is not the only iron oxidizer present. For example, we recovered 16S rRNA genes affiliated with WJ2, an iron-oxidizing taxon isolated from Wales (87). As with Ferrovaceae taxa, multiple co-occurring Fe(II) oxidizers may inhabit distinct niches that are not readily distinguishable in bulk samples. These co-occurring species may allow Fe(II) oxidation to proceed in a higher diversity of microenvironments and provide stability when geochemical conditions change (16). We propose that both intra- and interspecies diversity of co-occurring Fe(II) oxidizers contribute to the rapid Fe(II) oxidation rate. However, future culture-based efforts should be used to determine if or how diversity influences the Fe(II) oxidation rate. Effective bioremediation strategies should account for the potential of taxonomic and physiological diversity to enhance Fe(II) oxidation efficiency.

## Data Availability

Metagenomic reads are available on the NCBI Sequence Read Archive under accession number SRX18535481. Metagenome-assembled genomes are available under NCBI genome accession numbers JBVQOC000000000 (Wind_6), JBVQOD000000000 (Wind_5), and JBVQOE000000000 (Wind_11).

## SUPPLEMENTAL MATERIAL

Figure S1 aem.00700-26-s0001.pdf Full phylogeny of the Ferrovaceae based on the concatenated marker gene tree.

Table S1 aem.00700-26-s0002.xlsx Genomes used in the analysis, taxonomic classification, completeness, contamination, and strain heterogeneity of each genome; location and geochemical information; and presence or absence of relevant metabolic pathways and number of genes found that encode for flagellar proteins.

Table S2 aem.00700-26-s0003.xlsx Phototrophy-related genes present in Ca. *Solisferrum* genomes.

ASM does not own the copyrights to Supplemental Material that may be linked to, or accessed through, an article. The authors have granted ASM a non-exclusive, world-wide license to publish the Supplemental Material files. Please contact the corresponding author directly for reuse.
