# Supplementary figures and images for: Metabolic diversity of Ferrovaceae and potential contributions to iron oxidation
Source: Appl Environ Microbiol. 2026 Jun 3;92(7):e00700-26. doi: 10.1128/aem.00700-26 (PMC13390463; doi:10.1128/aem.00700-26)

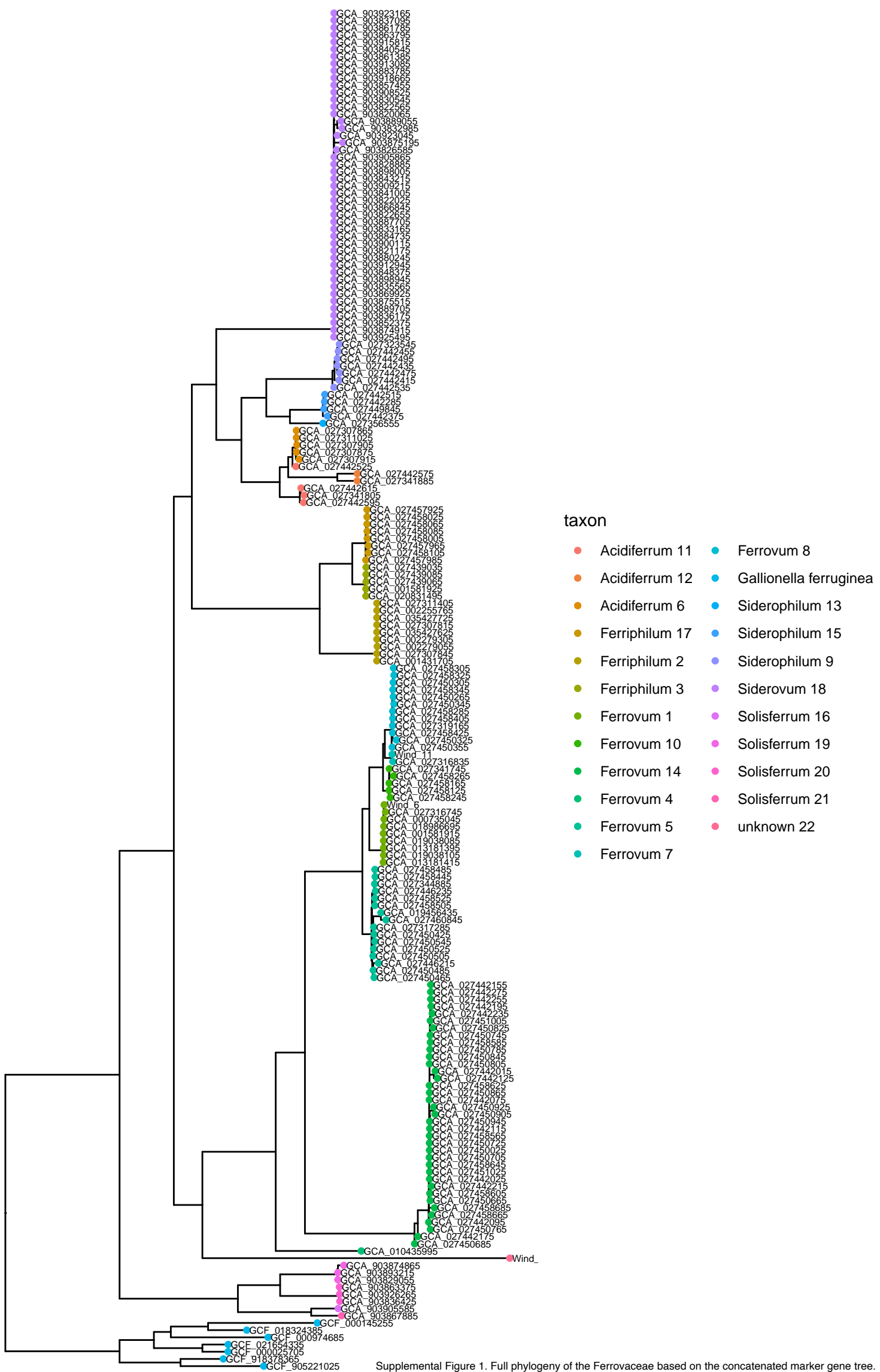

Supplement: Figure S1 — Full phylogeny of the Ferrovaceae based on the concatenated marker gene tree. [file aem.00700-26-s0001.pdf]
